# Supplementary material for: Admixture between old lineages facilitated contemporary ecological speciation in Lake Constance stickleback
Source: Nat Commun. 2019 Sep 18;10:4240. doi: 10.1038/s41467-019-12182-w (PMC6751218; doi:10.1038/s41467-019-12182-w)
Supplement: Supplementary file 1 — Supplementary Information [file 41467_2019_12182_MOESM1_ESM.pdf]

Supplementary Information

Admixture between old lineages facilitated contemporary ecological speciation in Lake Constance stickleback

Marques, Lucek et al.

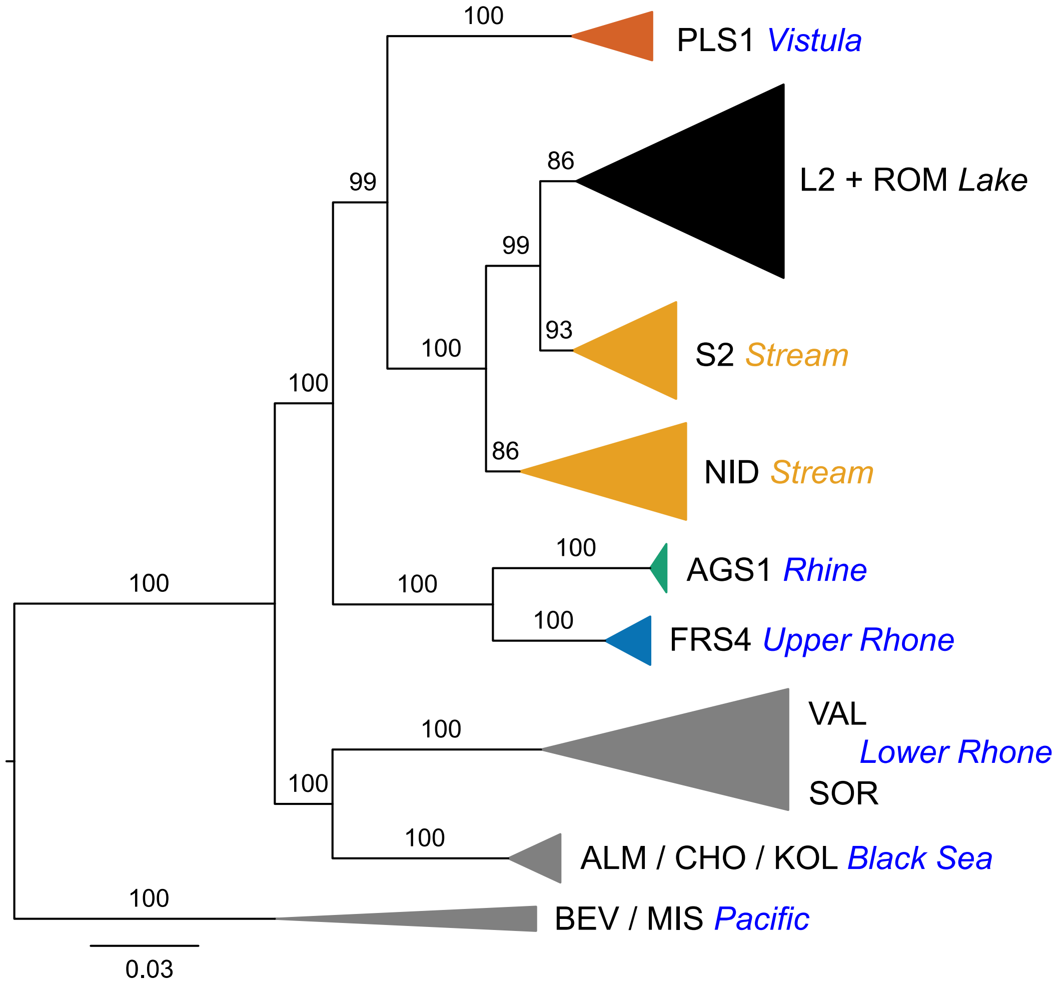

Supplementary Fig. 1 | Placement of Lake Constance stickleback in a phylogeographic context. Maximum-likelihood phylogeny based on concatenated autosomal SNPs embedding four Lake Constance populations into a European phylogeny consisting of divergent clades as shown in Fig. 1a. Note that (i) all Lake Constance populations together appear as one monophyletic clade, (ii) that individual lake populations are not separated, (iii) that stream stickleback populations are paraphyletic and (iv) that the inclusion of NID reduces the bootstrap support for the monophyly of the Lake Constance + East Europe clade, as expected if the population is of hybrid origin. Branch labels are percentage bootstrap support values. Source data are provided as a Source Data file.

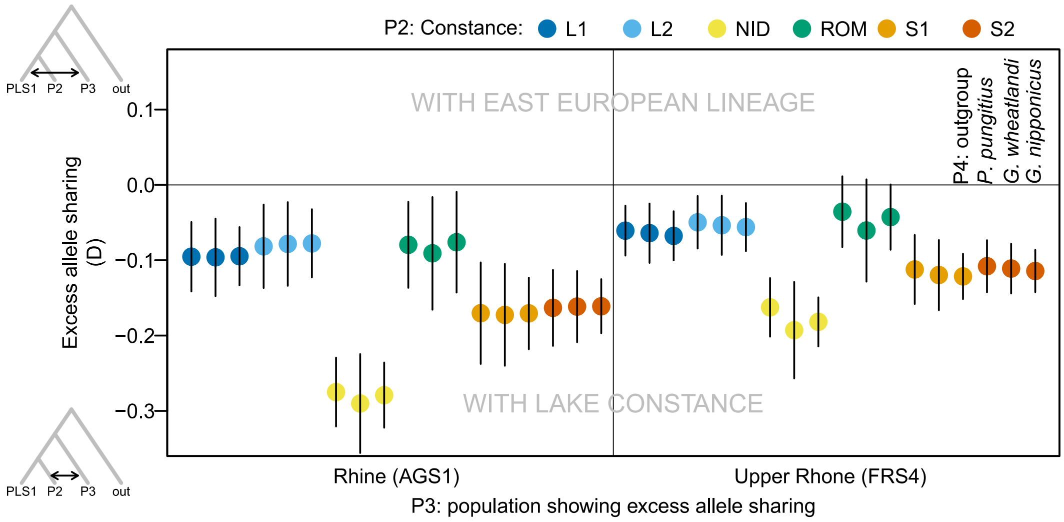

Supplementary Fig. 2 | Evidence for introgression of West European alleles into all Lake Constance populations. D-statistics show significant excess allele sharing of all Lake Constance population with West European lineages from the Rhine (AGS1) and the Upper Rhone (FRS4) compared to the more closely related East European lineage from the Vistula (PLS1). Significance is marginal to absent for lake populations (L1, L2, ROM), but strong for stream populations (S1, S2, NID). Colors indicate the Lake Constance population (P2: L1, L2, NID, S1, ROM, S2), while three neighboring points in turn use three different outgroups, ninespine stickleback (*Pungitius pungitius*), Black-spotted stickleback (*Gasterosteus wheatlandi*) and Japan Sea stickleback (*G. nipponicus*). Error bars indicate ±3 standard deviations around D-estimates. Source data are provided as a Source Data file.

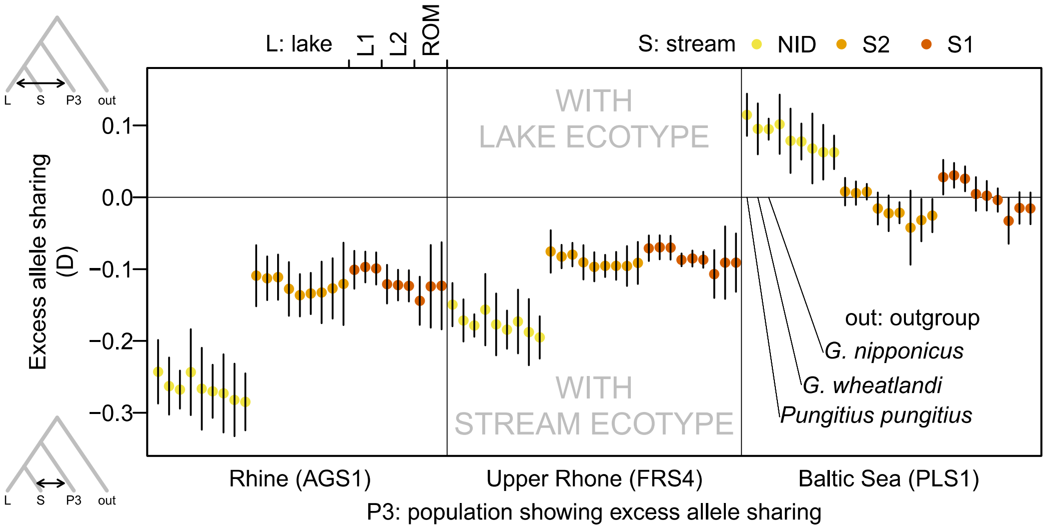

Supplementary Fig. 3 | Admixture evidence from additional lake populations and outgroups. Colors indicate the stream population (S: NID, S2, S1), three points in a row correspond to the same lake population (L: L1, L2, ROM), while three neighboring points in turn correspond to three different outgroups, ninespine stickleback (*Pungitius pungitius*), Black-spotted stickleback (*Gasterosteus wheatlandi*) and Japan Sea stickleback (*Gasterosteus nipponicus*). Error bars indicate ±3 standard deviations around D-estimates. Source data are provided as a Source Data file.

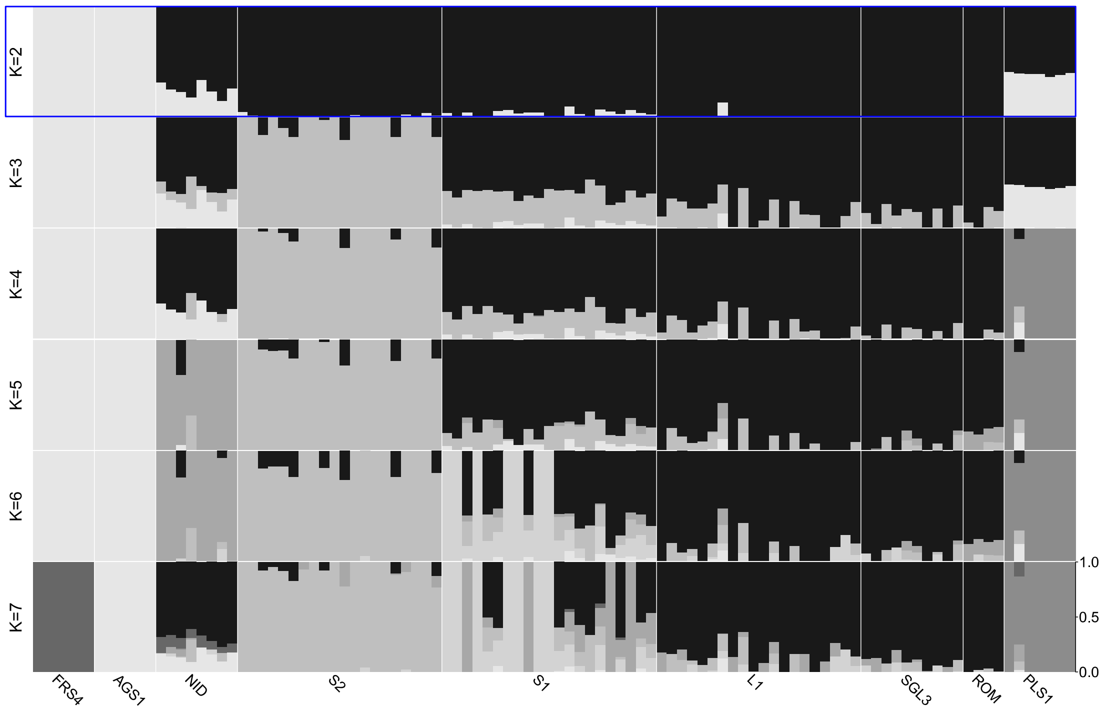

Supplementary Fig. 4 | Maximum-likelihood clustering of Lake Constance stickleback and European lineages (*SbfI* RAD-seq SNPs). The best number of clusters (*K*=2) as assessed from cross-validation is highlighted with a blue box. The y-axis shows the estimated cluster membership of each individual. Source data are provided as a Source Data file.

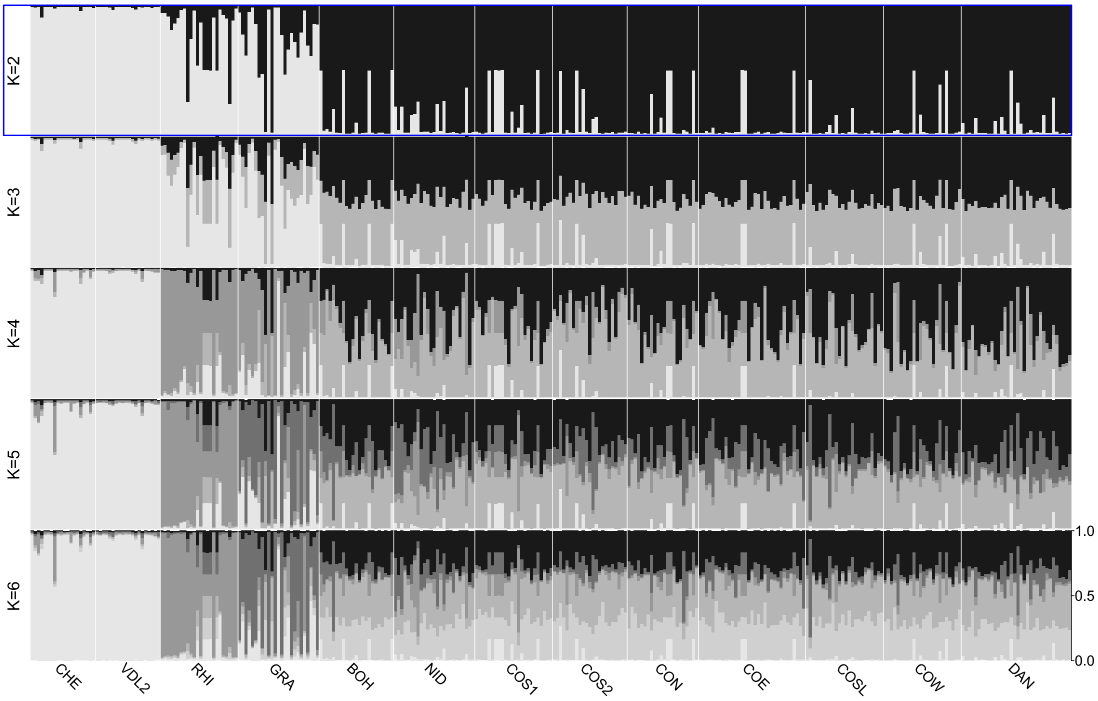

Supplementary Fig. 5 | Bayesian clustering of Lake Constance stickleback and European lineages (microsatellites). The best number of clusters (*K*=2) as assessed using the Evanno method (Evanno *et al.* 2005) is highlighted with a blue box. The y-axis shows the estimated cluster membership of each individual. Source data are provided as a Source Data file.

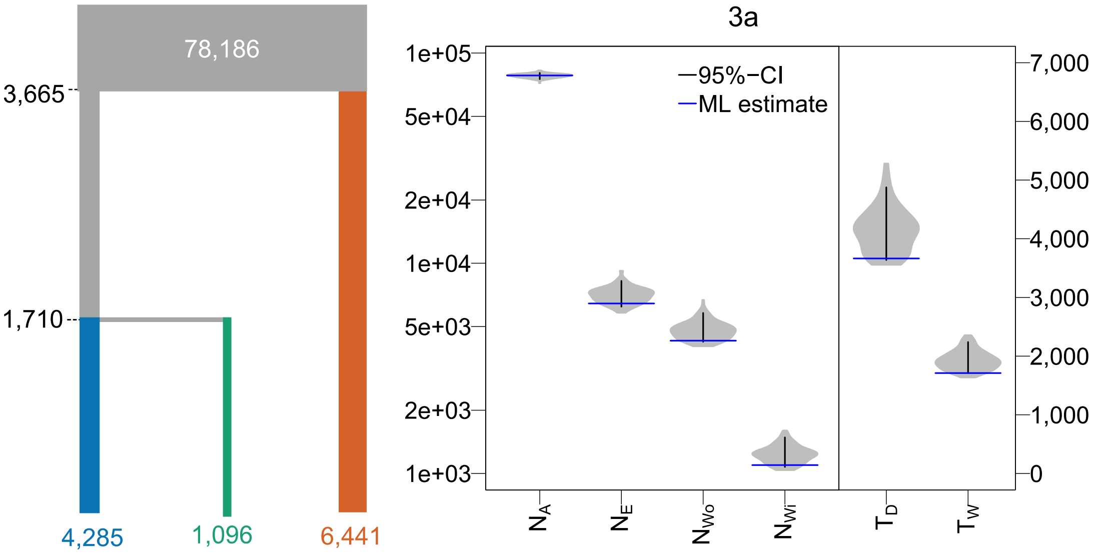

Supplementary Fig. 6 | Parameter estimates for model 3a. Maximum-likelihood parameter estimates for model 3a including West (Wi: Rhine, green; Wo: Rhone, blue) and East European (E: Vistula, red) populations. The left panel shows population sizes (colored, white numbers), and time estimates for population splits (black numbers). The right panel shows block-bootstrap parameter confidence intervals for the best models, with population sizes (N_i) in units of $2N_e$ and time (T_i) in numbers of generations. Maximum likelihood parameter estimates are indicated in blue and black error bars show 95%-confidence intervals (95%-CI). Source data are provided as a Source Data file.

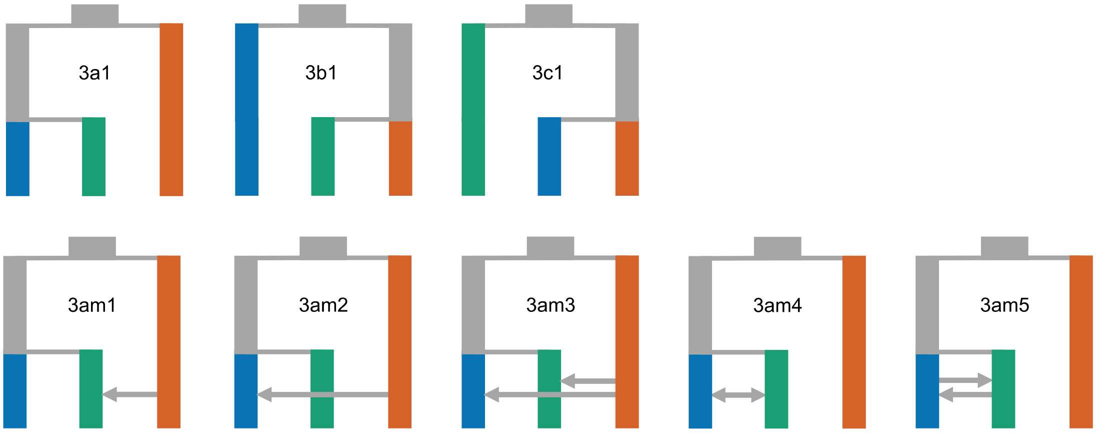

Supplementary Fig. 7 | Alternative demographic models tested. The first three models (3a1, 3b1, 3c1) introduce an additional parameter for the population size of the West and East European lineage ancestor, respectively, but did not considerably improve the likelihood (Supplementary Fig. 8). The latter five models test for admixture between the three ancestral lineages. While these more complex models performed equally well to the simpler model 3a without any admixture (Supplementary Fig. 8), the admixture proportions in all of these models were very close to zero (<0.5%) making them equivalent to model 3a, which all downstream modelling was based on (Fig. 2 b-d). Source data are provided as a Source Data file.

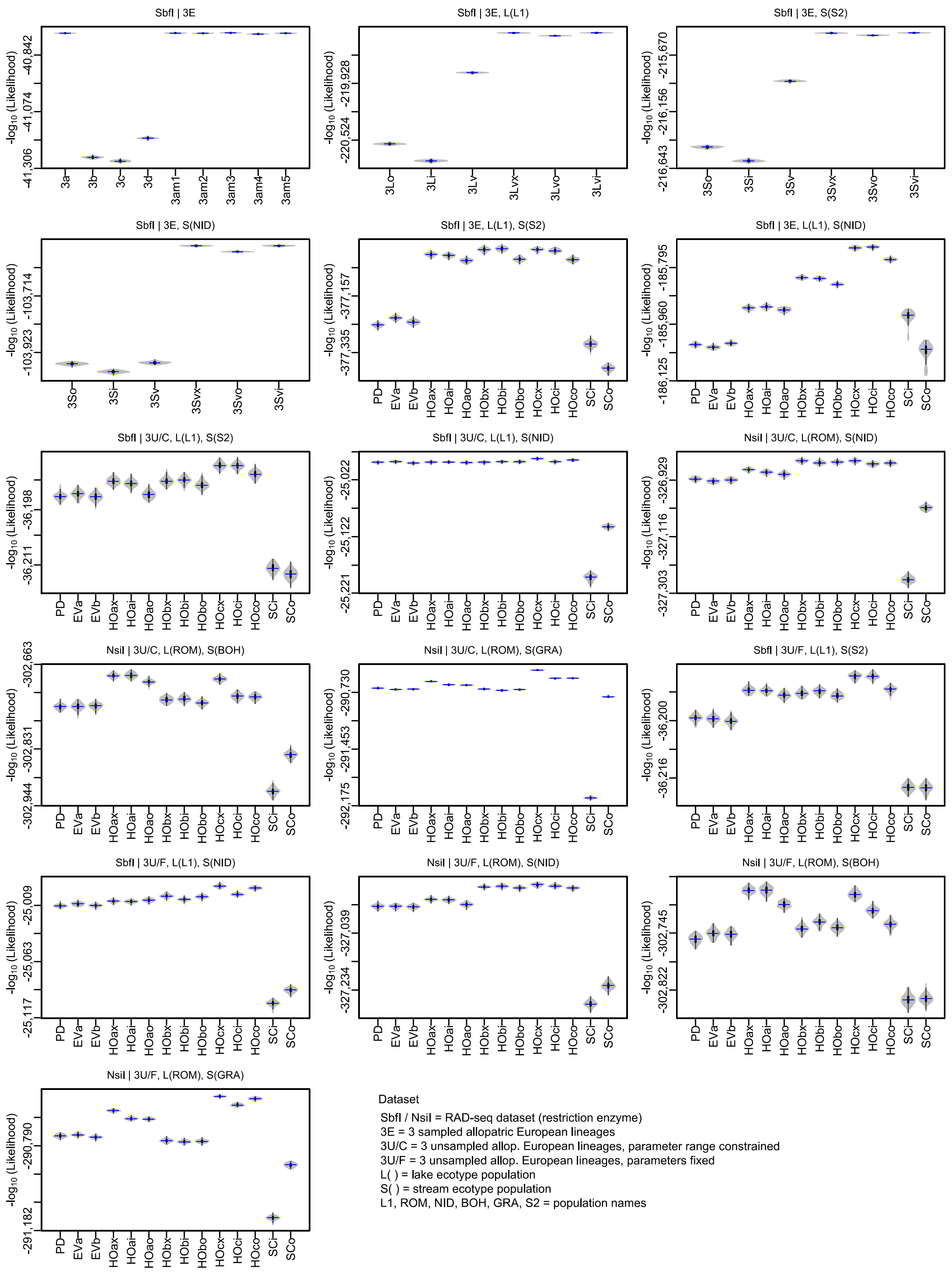

Supplementary Fig. 8 | Likelihood distributions of each tested demographic model. Violin plots show the distributions of $-\log_{10}$ likelihoods of 100 site-frequency spectra simulated under the maximum likelihood parameters inferred for each demographic model using the observed data. If likelihood distributions of two models overlap, this indicates that the two models fit the observed data similarly well. The best and similarly well-fitting models are both highlighted in Table 1. Blue horizontal bars indicate median likelihoods, black boxes delineate the 1st and 3rd quartile and error bars extend these by max. 1.5 times the interquartile range. Source data are provided as a Source Data file.

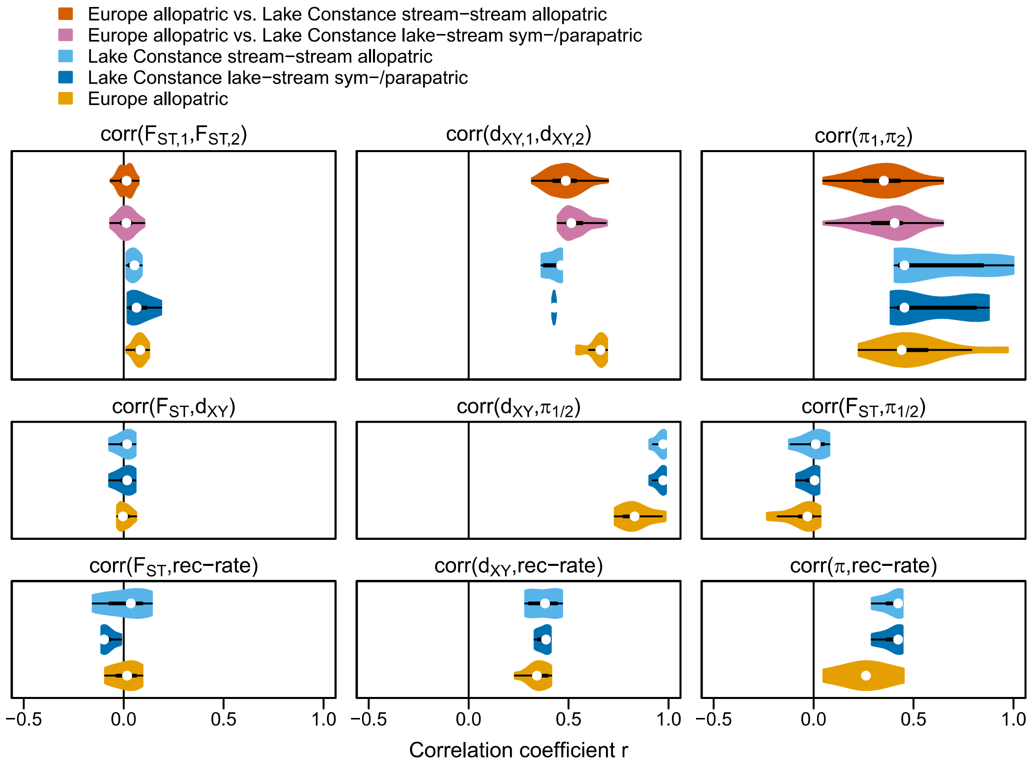

Supplementary Fig. 9 | Correlation of genome-wide differentiation (F_{ST}), diversity (π), divergence (d_{XY}) and recombination rate (cM Mbp^{-1}). Genome-wide differentiation is weakly correlated between allopatric populations, but not associated with recombination rate, suggesting that background selection does not play a major role in shaping differentiation. In Lake Constance, only differentiation between lake and stream ecotypes is negatively associated with recombination rate, but not differentiation between allopatric streams, suggesting that divergent selection between habitats rather than background selection drives differentiation. Shown are distributions of Pearson's correlation coefficients between statistics, for allopatric European population comparisons (yellow: AGS1, FRS4, PLS1, SOR, CHA), Lake Constance lake vs. stream ecotype comparisons (dark blue: L1 vs. S1 / S2, ROM vs. NID / GRA / BOH), Lake Constance stream vs. stream population comparisons (light blue: L1, S2, NID, GRA, BOH) and for allopatric European populations vs. Lake Constance lake vs. stream ecotypes or stream vs. stream populations (pink, red). Only comparisons of non-overlapping population pairs, e.g. $F_{ST(AGS1,FRS4)}$ vs. $F_{ST(PLS1,SOR)}$ are shown for F_{ST} and d_{XY} comparisons to avoid pseudo-replication. Statistics were computed in windows of >2,500 sequenced base pairs. Mean recombination rate per window was estimated at 10 regularly spaced positions in each window. Black box plots delineate the 1st and 3rd quartile, with whiskers extending these by max. 1.5 times the interquartile range. The white dot shows the median. Source data are provided as a Source Data file.

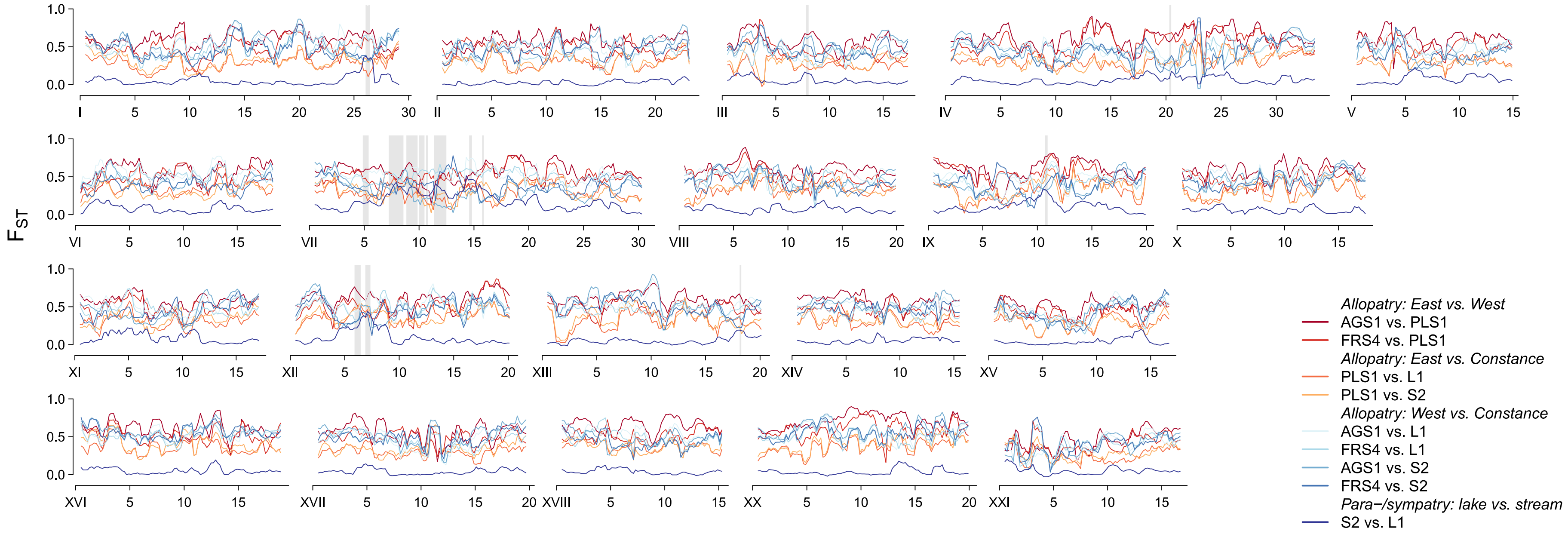

Supplementary Fig. 10 | Genomic differentiation between Lake Constance lake (L1) and stream (S2) ecotypes, West European and East European lineages. Shown are weighted mean F_{ST} in 1 Mbp sliding window with 200 kb step size for different population comparisons. Chromosome numbers are shown at the bottom left of each x-axis, x-axis values give positions in Mbp, grey boxes indicate genomic islands of parallel differentiation (Marques *et al.* 2016). Source data are provided as a Source Data file.

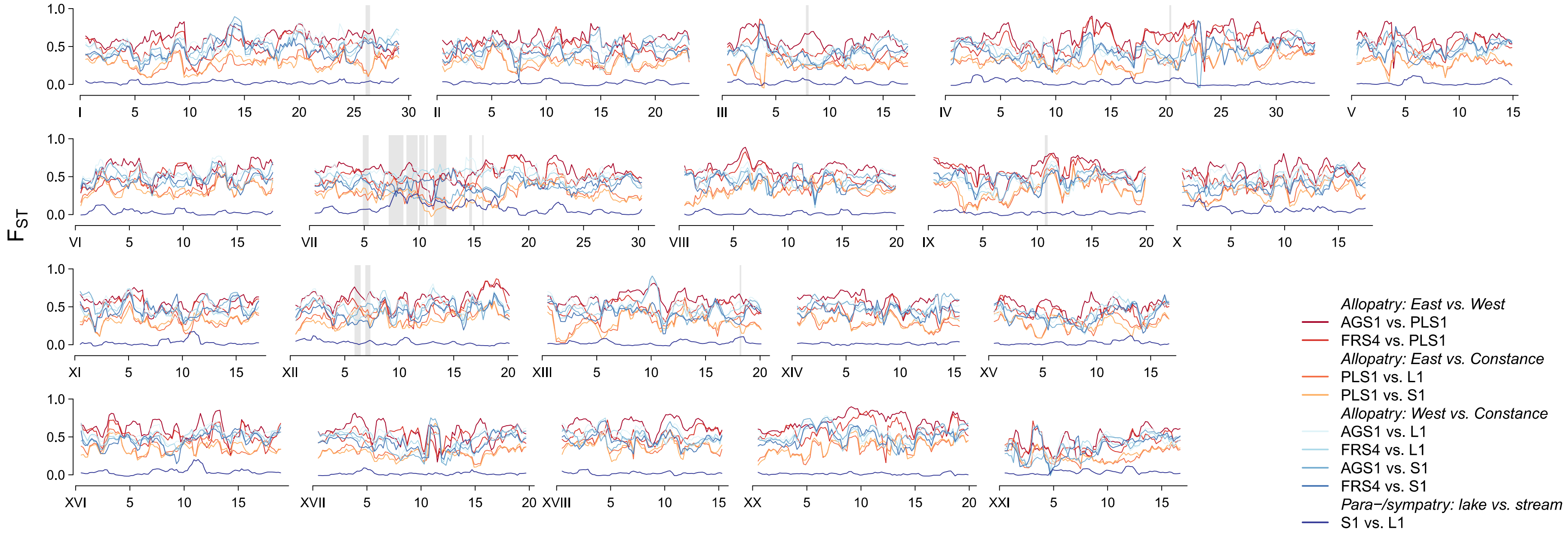

Supplementary Fig. 11 | Genomic differentiation between Lake Constance lake (L1) and stream (S1) ecotypes, West European and East European lineages. Shown are weighted mean F_{ST} in 1 Mbp sliding window with 200 kb step size for different population comparisons. Chromosome numbers are shown at the bottom left of each x-axis, x-axis values give positions in Mbp, grey boxes indicate genomic islands of parallel differentiation (Marques *et al.* 2016). Source data are provided as a Source Data file.

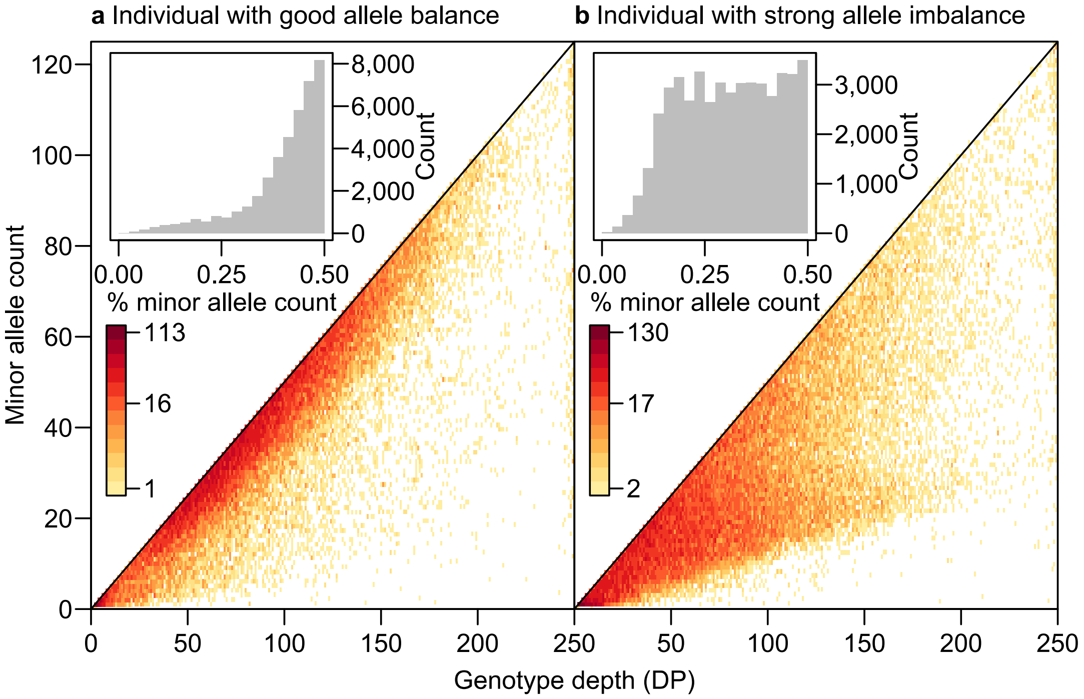

Supplementary Fig. 12 | Different levels of allele imbalance in two individuals of the *NsiI* dataset. Allele imbalance is an indicator of PCR errors, contamination or other artefacts. PCR errors can have a strong effect on the site frequency spectrum, in particular singletons – we thus excluded individuals with strong allele imbalance (b) and only included individuals with good balance between major and minor alleles (a).

Supplementary Table 1 | List of populations and sample sizes for each data type. Superscript letters give the references to where the data is published.

ID	Location	Coordinates N/E	Catchment	Habitat	mtDNA	µsat	Plate Morph	*SbfI* RAD	*NsiI* RAD	*PstI* RAD
L1	Altenrhein, CH	47º29'08"/9º32'37"	Lake Constance, Rhine, North Sea	lake	22[c]	-	20[k]	20[k]	-	-
L2	Marina Rheinhof, CH	47º29'55"/9º33'25"	Lake Constance, Rhine, North Sea	lake	-	-	10[k]	10[k]	-	-
S1	Buriet, CH	47º28'43"/9º33'30"	Lake Constance, Rhine, North Sea	stream	6[c]	-	17[k]	21[k]	-	-
S2	Oberried, CH	47º28'43"/9º33'30"	Lake Constance, Rhine, North Sea	stream	20[c]	-	20[k]	20[k]	-	-
BOH	Iznang, DE	47º19'38"/9º34'24"	Lake Constance, Rhine, North Sea	stream	20[e]	23[e]	20[e]	-	10[j]	-
CON	Bohlingen, DE	47º28'43"/9º33'30"	Lake Constance, Rhine, North Sea	lake	22[e]	22[e]	21[e]	-	-	-
GRA	Grasbeuren, DE	47º43'39"/9º18'23"	Lake Constance, Rhine, North Sea	stream	25[e]	25[e]	25[e]	-	9[j]	-
COE	Mühlhofen, DE	47º44'11"/9º15'49"	Lake Constance, Rhine, North Sea	lake	18[e]	33[e]	25[e]	-	-	-
NID	Niederaach, CH	47º28'43"/9º33'30"	Lake Constance, Rhine, North Sea	stream	33[b,e]	25[b,e]	24[e]	8[f]	15[j]	-
ROM	Romanshorn, CH	47º33'22"/9º22'48"	Lake Constance, Rhine, North Sea	lake	21[b,e]	24[b,e]	21[e]	4[f]	7[j]	-
COS	Fussach, AT	47º29'29"/9º39'40"	Lake Constance, Rhine, North Sea	lake	25[b,e]	24[b,e]	23[e]	-	-	-
COS1	Hohenems, AT	47º21'18"/9º40'10"	Lake Constance, Rhine, North Sea	stream	25[b,e]	24[e]	25[e]	-	-	-
COS2	Rankweil, AT	47º16'19"/9º35'32"	Lake Constance, Rhine, North Sea	stream	22[e]	23[b,e]	24[e]	-	-	-
DAN	Kirchbierlingen, DE	48º14'04"/9º43'30"	Danube, Black Sea	stream	29[e]	34[e]	24[e]	-	-	-
DKM3	Fanø, DK	55º24'15"/8º24'26"	North Sea	marine	5*[,c]	-	-	-	-	-
PLS1	Broszkowice, PL	50º03'13"/19º14'46"	Vistula, Baltic Sea	stream	3*	-	20[i]	7*	-	-
BUG	Western Bug, PL	52º55'00"/23º34'00"	Vistula, Baltic Sea	stream	1[a]	-	-	-	-	-
AGS1	Möhlin, CH	47º34'40"/7º50'17"	Rhine, North Sea	stream	12[c]	-	25[c]	6*	-	-
RHI	Basel, CH	47º32'44"/7º33'51"	Rhine, North Sea	stream	22[e]	24[e]	-	-	-	-
VDL2	St. Sulpice, CH	46º31'02"/6º34'41"	Lake Geneva, Rhone	lake	16[b,c]	20[a]	-	-	-	-
VDL6	Les Grangettes, CH	46º23'45"/6º53'18"	Lake Geneva, Rhone	lake	-	-	70[h] (historic)	-	-	-
CHE	Chessel, CH	46º20'52"/6º54'37"	Lake Geneva, Rhone	stream	-	20[b]	-	-	-	-
VSS1	Sion, CH	46º12'50"/7º18'53"	Lake Geneva, Rhone	stream	19[c]	-	-	-	-	-
FRS4	Cormoz, FR	45º58'03"/5º17'39"	Rhone, Mediterranean	stream	-	-	32[i]	6*	-	-
SEY	Saymard, FR	45º58'00"/5º18'00"	Rhone, Mediterranean	stream	1[a]	-	-	-	-	-
RUD	Ru du Gua, FR	45º50'00"/5º15'00"	Rhone, Mediterranean	stream	3[a]	-	-	-	-	-
FOR	Formans, FR	45º27'00"/4º46'00"	Rhone, Mediterranean	stream	2[a]	-	-	-	-	-
VAL	Valence FR	44º56'02"/4º53'08"	Rhone, Mediterranean	stream	-	-	-	6*	-	-
SOR	Sorgue FR	43º54'34"/5º05'10"	Rhone, Mediterranean	stream	1[a]	-	20[i]	6*	-	-
CHA	Chamoux FR	47º27'24"/3º39'13"	Seine, Atlantic	stream	1[a]	-	-	6*	-	-
FRS11	Saint-Pourcain-sur-Sioule, FR	46º17'56"/3º17'00"	Allier, Atlantic	stream	-	-	-	6*	-	-
NER	Neretva BA	43º05'57"/17º42'58"	Adriatic, Mediterranean	stream	1[a]	-	-	-	-	-
SKA	Skadar ME	42º14'00"/19º06'00"	Adriatic, Mediterranean	lake	1[a]	-	-	-	-	-
MAN	SE-Romania, RO	43º38'00"/28º29'00"	Black Sea	marine	2[a]	-	-	-	-	-
ALM	River Alma, UA	44º50'00"/33º36'00"	Black Sea	stream	2[a]	-	-	-	-	1[l]
CHO	River Chornaya, UA	44º34'00"/33º28'00"	Black Sea	stream	-	-	-	-	-	1[l]
KOL	River Kolanraes, UA	46º13'00"/33º41'00"	Black Sea	stream	-	-	-	-	-	1[l]
MIS	Misty Lake BC, CA	50º36'20"/127º15'46" W	Pacific	lake	-	-	-	-	-	1[l]
BEV	Beaver Lake BC, CA	50º35'59"/127º19'11" W	Pacific	lake	-	-	-	-	-	1[l]

References. a Makinen and Merila (2008), b Berner *et al.* (2009), c Lucek *et al.* (2010), d Lucek *et al.* (2012), e Moser *et al.* (2012), f Roesti *et al.* (2012), g Lucek *et al.* (2013), h Lucek *et al.* (2014), i Lucek and Seehausen (2015), j Roesti *et al.* (2015), k Marques *et al.* (2016), l Fang *et al.* (2018), *new data, GenBank accessions MN082769-MN082781, Sequence Read Archive (SRA) accessions SRR9317386-SRR9317452, SRR9335375-SRR9335380, SRA-BioProject accession PRJNA549360.

Supplementary Table 2 | Fit of additional demographic models and datasets. $\log_{10}$ likelihood difference between observed and expected site-frequency spectra (ΔLL) and difference in Akaike information criterion (ΔAIC) between the best and all models for a given dataset are shown. Best-fitting models and models with very similar likelihood are marked with an asterisk. For all five population models, the three sister lineages were modeled as unsampled (‘ghost’: º) populations with parameter search ranges for these sister populations either constrained to 95% confidence intervals of model 3a or parameters fixed to the maximum likelihood estimates of model 3a (see Supplementary Fig. 6). Source data are provided as a Source Data file.

Model	Dataset	ΔLL	ΔAIC
3a1*	FRS4 AGS1 PLS1 (SbfI)	80	-13*
3b1		510	-1,991
3c1		511	-1,999
3am1*		77	0*
3am2*		80	-13*
3am3*		78	-7*
3am4*		82	-24*

Dataset	3 unsampled sisters (constrained), L1, S2 (*SbfI*)		3 unsampled sisters (constrained), L1, NID (*SbfI*)		3 unsampled sisters (fixed), L1, S2 (*SbfI*)		3 unsampled sisters (fixed), L1, NID (*SbfI*)	
Model	ΔLL	ΔAIC	ΔLL	ΔAIC	ΔLL	ΔAIC	ΔLL	ΔAIC
PD	22	-21	57	-23	26	-45	73	-73
EVa	21	-21	56	-18	26	-47	72	-68
EVb	21	-21	58	-32	27	-51	73	-76
HOax	19	-16	55	-19	19	-15	70	-63
HOai	19	-13	57	-25	19	-16	71	-66
HOao	20	-19	57	-25	21	-22	69	-60
HObx	19	-15	55	-21	20	-20	63	-34
HObi	19	-13	54	-13	19	-13	66	-46
HObo	19	-15	55	-18	20	-18	64	-38
HOcx*	15	-4*	51	-7*	15	-5*	55	-1*
HOci*	15	0*	54	-18	15	0*	63	-35
HOco*	18	-12	50	0*	19	-19	55	0*
SCi	39	-102	252	-921	45	-130	167	-504
SCo	39	-104	166	-526	45	-129	155	-452

Dataset	3 unsampled sisters (constrained), ROM / NID (*NsiI*)º		3 unsampled sisters (constrained), ROM / BOH (*NsiI*)º		3 unsampled sisters (constrained), ROM / GRA (*NsiI*)º	
Model	ΔLL	ΔAIC	ΔLL	ΔAIC	ΔLL	ΔAIC
PD	169	-298	152	-265	349	-1,020
EVa	172	-314	149	-257	363	-1,087
EVb	163	-275	147	-247	366	-1,100
HOax*	131	-131	93	0*	273	-675
HOai*	139	-166	95	-10*	307	-830
HOao*	144	-188	105	-55*	311	-847
HObx*	105	-10*	139	-212	366	-1,105
HObi*	111	-34*	135	-193	377	-1,153
HObo*	109	-26*	142	-224	366	-1,102
HOcx*	102	0*	102	-50*	125	0*
HOci*	112	-44*	130	-172	227	-466
HOco*	108	-26*	136	-200	220	-433
SCi	498	-1,814	319	-1,035	1739	-7,419
SCo	255	-693	244	-693	453	-1,500

Supplementary Table 3 | Test for deviations of genomic island and genome-wide differentiation between Lake Constance and allopatric European populations. Results from a permutation test with 10,000 permutations of the position of genomic islands of ecotype differentiation in the genome. A minus sign indicates cases in which genome-wide differentiation exceeds genomic island differentiation. Significant deviations suggest that stream populations South-East of Lake Constance have been introgressed with West European alleles, independent of whether a Rhine or a Rhone population is used for the test. Significance levels are ns: $p \geq 0.05$, * $p < 0.05$, ** $p < 0.01$. See Supplementary Table 1 for population codes.

Lineage	vs. Constance lake		vs. Constance stream	
West Europe (Rhone)	FRS4 vs. L1	ns	FRS4 vs. S1	-*
			FRS4 vs. S2	-**
West Europe (Rhine)	AGS1 vs. L1	ns	AGS1 vs. S1	-**
			AGS1 vs. S2	-**
East Europe (Baltic Sea)	PLS1 vs. L1	ns	PLS1 vs. S1	ns
			PLS1 vs. S2	ns

Supplementary References

Berner D, Grandchamp AC, Hendry AP (2009) Variable progress toward ecological speciation in parapatry: stickleback across eight lake-stream transitions. *Evolution* **63**, 1740-1753.

Evanno G, Regnaut S, Goudet J (2005) Detecting the number of clusters of individuals using the software STRUCTURE: a simulation study. *Mol Ecol* **14**, 2611-2620.

Fang B, Merila J, Ribeiro F, Alexandre CM, Momigliano P (2018) Worldwide phylogeny of three-spined sticklebacks. *Mol Phylogenet Evol* **127**, 613-625.

Lucek K, Lemoine M, Seehausen O (2014) Contemporary ecotypic divergence during a recent range expansion was facilitated by adaptive introgression. *Journal of Evolutionary Biology* **27**, 2233-2248.

Lucek K, Roy D, Bezault E, Sivasundar A, Seehausen O (2010) Hybridization between distant lineages increases adaptive variation during a biological invasion: stickleback in Switzerland. *Mol Ecol* **19**, 3995-4011.

Lucek K, Seehausen O (2015) Distinctive insular forms of threespine stickleback (*Gasterosteus aculeatus*) from western Mediterranean islands. *Conservation Genetics* **16**, 1319-1333.

Lucek K, Sivasundar A, Roy D, Seehausen O (2013) Repeated and predictable patterns of ecotypic differentiation during a biological invasion: lake-stream divergence in parapatric Swiss stickleback. *J Evol Biol* **26**, 2691-2709.

Lucek K, Sivasundar A, Seehausen O (2012) Evidence of adaptive evolutionary divergence during biological invasion. *PLoS One* **7**, e49377.

Makinen HS, Merila J (2008) Mitochondrial DNA phylogeography of the three-spined stickleback (*Gasterosteus aculeatus*) in Europe - evidence for multiple glacial refugia. *Mol Phylogenet Evol* **46**, 167-182.

Marques DA, Lucek K, Meier JI*, et al.* (2016) Genomics of rapid incipient speciation in sympatric threespine stickleback. *PLoS Genet* **12**, e1005887.

Moser D, Roesti M, Berner D (2012) Repeated lake-stream divergence in stickleback life history within a Central European lake basin. *PLoS One* **7**, e50620.

Roesti M, Kueng B, Moser D, Berner D (2015) The genomics of ecological vicariance in threespine stickleback fish. *Nat Commun* **6**, 8767.

Roesti M, Salzburger W, Berner D (2012) Uninformative polymorphisms bias genome scans for signatures of selection. *BMC Evol Biol* **12**, 94.
