# Supplementary material for: Admixture between old lineages facilitated contemporary ecological speciation in Lake Constance stickleback
Source: Nat Commun. 2019 Sep 18;10:4240. doi: 10.1038/s41467-019-12182-w (PMC6751218; doi:10.1038/s41467-019-12182-w)
Supplement: Supplementary file 3 — Reporting Summary [file 41467_2019_12182_MOESM3_ESM.pdf]

nature research

Corresponding author(s): Ole Seehausen

Last updated by author(s): Aug 13, 2019

Reporting Summary

Nature Research wishes to improve the reproducibility of the work that we publish. This form provides structure for consistency and transparency in reporting. For further information on Nature Research policies, see Authors & Referees and the Editorial Policy Checklist .

Statistics

For all statistical analyses, confirm that the following items are present in the figure legend, table legend, main text, or Methods section.

n/a	Confirmed	
☐	☒	The exact sample size (n) for each experimental group/condition, given as a discrete number and unit of measurement
☐	☒	A statement on whether measurements were taken from distinct samples or whether the same sample was measured repeatedly
☐	☒	The statistical test(s) used AND whether they are one- or two-sided<br>*Only common tests should be described solely by name; describe more complex techniques in the Methods section.*
☐	☒	A description of all covariates tested
☐	☒	A description of any assumptions or corrections, such as tests of normality and adjustment for multiple comparisons
☐	☒	A full description of the statistical parameters including central tendency (e.g. means) or other basic estimates (e.g. regression coefficient) AND variation (e.g. standard deviation) or associated estimates of uncertainty (e.g. confidence intervals)
☐	☒	For null hypothesis testing, the test statistic (e.g. F, t, r) with confidence intervals, effect sizes, degrees of freedom and P value noted<br>*Give P values as exact values whenever suitable.*
☐	☒	For Bayesian analysis, information on the choice of priors and Markov chain Monte Carlo settings
☐	☒	For hierarchical and complex designs, identification of the appropriate level for tests and full reporting of outcomes
☐	☒	Estimates of effect sizes (e.g. Cohen's d, Pearson's r), indicating how they were calculated

Our web collection on statistics for biologists contains articles on many of the points above.

Software and code

Policy information about availability of computer code

Data collection	No software was used for data collection.
Data analysis	Software used and versions are indicated in the Methods section. All custom scripts mentioned in the Methods section have been deposited and are available on GitHub under https://github.com/marqueda.

For manuscripts utilizing custom algorithms or software that are central to the research but not yet described in published literature, software must be made available to editors/reviewers. We strongly encourage code deposition in a community repository (e.g. GitHub). See the Nature Research guidelines for submitting code & software for further information.

Data

Policy information about availability of data

All manuscripts must include a data availability statement. This statement should provide the following information, where applicable:

- Accession codes, unique identifiers, or web links for publicly available datasets
- A list of figures that have associated raw data
- A description of any restrictions on data availability

The source data underlying Figs. 1-6, Tab. 1, Supplementary Figs. S1-S11 and Tab. S1 are provided as a Source Data file. Sequence data has been deposited on GenBank under accessions MN082769-MN082781 and on Sequence Read Archive under accessions SRR9317386-SRR9317452, SRR9335375-SRR9335380 and SRA-BioProject accession PRJNA549360. Table S3 summarizes sources of previously published, utilized data.

Field-specific reporting

Please select the one below that is the best fit for your research. If you are not sure, read the appropriate sections before making your selection.

Ecological, evolutionary & environmental sciences study design

All studies must disclose on these points even when the disclosure is negative.

Study description	Biogeographic reconstruction of the origin and evolution of Lake Constance stickleback, using phenotypic, genomic and genetic data and phylogenomic, population genomic analysis and demographic modelling.
Research sample	Threespine stickleback (Gasterosteus aculeatus), broad biogeographic sampling of watersheds surrounding the geographic area around Lake Constance including catchments draining into the Atlantic, Black Sea, Baltic Sea, North Sea and Mediterranean.
Sampling strategy	Sampling of at least 6 individuals per population for genomic analysis, as outlined in Table S3. Combination of new data and previously analyzed datasets - see there for details.
Data collection	Stickleback were caught in the field as described in the methods section by collaborators in France.
Timing and spatial scale	Spatial and time scale are described in Table S3 and references therein.
Data exclusions	Reads from libraries GQIv13a/b were excluded from the computation of site-frequency spectra for demographic modelling purposes, due to high levels of PCR duplicates and PCR errors.
Reproducibility	No attempts to repeat the experiment have been conducted yet.
Randomization	For the computation of site-frequency spectra, we subsampled a number of fixed genotypes randomly from the available non-missing genotypes, without replacement.
Blinding	Blinding was not relevant for our genomic study, given that no observer-biased measurements were taken.

Did the study involve field work? ☒ Yes ☐ No

Field work, collection and transport

Field conditions	Stickleback were collected as adults in the breeding season (June-July), with details listed in Table S3 and references therein.
Location	See Table S3.
Access and import/export	Fish were collected in three streams in France by members of the departmental federations for fisheries, under their continuing permission to conduct scientific fisheries using techniques such as electrofishing. No import / export permits were required for preserved fish specimens.
Disturbance	Disturbances of other fish species through electrofishing was limited to 1-2 hours on a single day at each sampling site.

Reporting for specific materials, systems and methods

We require information from authors about some types of materials, experimental systems and methods used in many studies. Here, indicate whether each material, system or method listed is relevant to your study. If you are not sure if a list item applies to your research, read the appropriate section before selecting a response.

Materials & experimental systems

n/a	Involved in the study	
☒	☐	Antibodies
☒	☐	Eukaryotic cell lines
☒	☐	Palaeontology
☐	☒	Animals and other organisms
☒	☐	Human research participants
☒	☐	Clinical data

Methods

n/a	Involved in the study	
☒	☐	ChIP-seq
☒	☐	Flow cytometry
☒	☐	MRI-based neuroimaging

Animals and other organisms

Policy information about studies involving animals; ARRIVE guidelines recommended for reporting animal research

Laboratory animals	This study did not involve any laboratory animals.
Wild animals	Populations of threespine stickleback Gasterosteus aculeatus were captured in three streams in France, by members of the departmental federations for fisheries, under their continuing permission to conduct scientific fisheries using electrofishing as capturing technique. Fish were euthanized in the field with an overdose of clove oil or MS-222 in accordance with the respective

fisheries regulations.

Field-collected samples	This study did not involve live field-collected samples.
Ethics oversight	No ethical approval or guidance was required for this study.

Note that full information on the approval of the study protocol must also be provided in the manuscript.
